# Clinical Implications and Prognostic Values of *Prostate Cancer Susceptibility Candidate* Methylation in Primary Nonmuscle Invasive Bladder Cancer

**DOI:** 10.1155/2015/402963

**Published:** 2015-05-13

**Authors:** Young-Won Kim, Hyung-Yoon Yoon, Sung Pil Seo, Sang Keun Lee, Ho Won Kang, Won Tae Kim, Heui Je Bang, Dong Hee Ryu, Seok-Joong Yun, Sang-Cheol Lee, Wun-Jae Kim, Yong-June Kim

**Affiliations:** ^1^Department of Urology, College of Medicine, Chungbuk National University, Cheongju 362-763, Republic of Korea; ^2^Department of Rehabilitation, College of Medicine, Chungbuk National University, Cheongju 362-763, Republic of Korea; ^3^Department of Surgery, College of Medicine, Chungbuk National University, Cheongju 362-763, Republic of Korea

## Abstract

DNA methylation is the most common and well-characterized epigenetic change in human cancer. Recently, an association between *prostate cancer susceptibility candidate* (*PRAC*) methylation and genitourinary cancer was proposed. The aim of the present study was to evaluate the association between *PRAC* methylation status and clinicopathological parameters and prognosis in long-term follow-up primary nonmuscle invasive bladder cancer (NMIBC). The clinical relevance of *PRAC* methylation was determined in 136 human bladder specimens (eight normal controls [NCs] and 128 primary NMIBCs) using quantitative pyrosequencing analysis. *PRAC* methylation was significantly higher in NMIBC patients than in NCs and was significantly associated with higher grade and more advanced stage of cancer. Kaplan-Meier estimates revealed significant difference in tumor recurrence and progression according to *PRAC* methylation status (both *p* < 0.05). Multivariate Cox regression analysis revealed that the *PRAC* methylation status was a strong predictor of recurrence (hazard ratio [HR], 2.652; *p* = 0.012) and progression (HR, 9.531; *p* = 0.035) of NMIBC. Enhanced methylation status of *PRAC* was positively associated with a high rate of recurrence and progression in NMIBC patients, suggesting that *PRAC* methylation may be a promising prognostic marker of NMIBC.

## 1. Introduction

Because bladder cancer is a heterogeneous disease, pathologically similar tumors may behave differently. Numerous factors are likely involved in disease outcomes, and many patients with nonmuscle invasive bladder cancer (NMIBC) experience disease recurrence and progression after primary treatment [1, 2]. Therefore, identifying patients at high risk of recurrence and progression who would benefit from more aggressive treatment, as well as those at low risk who require less intensive surveillance after initial adequate therapy, is challenging. Currently, conventional clinicopathological factors are insufficient to predict the outcome of patients with NMIBC. Thus, additional biomarkers are needed to predict the prognosis of NMIBC patients.

As in most other human cancers, bladder cancer originates from multiple combinations of genetic, epigenetic, and environmental factors [3–6]. DNA methylation, which inactivates tumor suppressor genes, is the most common and well-characterized epigenetic change in human cancer and may be a potential biomarker for cancer [6, 7]. Recent research proposed an association between the* prostate cancer susceptibility candidate* (*PRAC*) gene and human tumors, including prostate and renal cell carcinoma (RCC) [8–12]. In particular,* PRAC* hypermethylation is the hallmark methylation phenotype in RCC, and its alterations in precancerous stages may be associated with tumor aggressiveness and patient prognosis [11, 12].

To our knowledge, no study has evaluated the prognostic role of the* PRAC* gene in bladder cancer. The aim of the present study was to evaluate the impact of* PRAC* methylation status on clinicopathological parameters and prognosis in long-term follow-up primary NMIBC patients.

## 2. Materials and Methods

### 2.1. Subjects and Sample Collection

A total of 136 human bladder tissues (eight normal controls [NC] and 128 NMIBC) were used for pyrosequencing (PSQ) analyses (Table 1). We selected available bladder tissues from Biobank which was utilized in our previous study [13]. NMIBC tissues were obtained from primary NMIBC patients who underwent transurethral resection (TUR) for histologically diagnosed transitional cell carcinomas between 1995 and 2010 at our institute. To exclude the possibility of incomplete resection or factors that may affect analyses, patients who were followed for less than 6 months or those that experienced disease relapse within 6 months were excluded from this study. NC tissues were obtained from individuals with benign prostate hyperplasia or bladder injury.

All tumors were macrodissected within 15 minutes of surgical resection. Each NMIBC specimen was confirmed by pathological analysis of a section of the tissue that was obtained from the TUR specimens, immediately frozen in liquid nitrogen, and stored at −80°C. The specimens were provided by the Chungbuk National University Hospital, a member of the National Biobank of Korea, which is supported by the Ministry of Health, Welfare, and Family Affairs. The collection and analysis of all samples were approved by the Chungbuk National University Hospital Institutional Review Board (GR2010-12-010), and informed consent was obtained from each subject.

Tumor staging was classified according to the 2002 TNM classification and the 1973 World Health Organization grading systems [14]. A second TUR was performed 2–4 weeks after the initial resection if a bladder cancer specimen did not include proper muscle or if a high-grade tumor was detected. Patients with intermediate- or high-risk NMIBC received one cycle of intravesical instillation therapy. Each patient was followed up and managed according to standard recommendations [1, 2]. Recurrence was defined as the return of primary NMIBC at a lower or equivalent pathologic stage (Ta/T1), and progression was defined as muscular invasion (TNM stage T2 or higher) or nodal/distant metastatic disease.

### 2.2. DNA Extraction and PSQ Analysis

Genomic DNA was extracted by standard methods using the Wizard Genomic DNA Purification System (Promega). Bisulfite conversion of genomic DNA was carried out using the EZ DNA Methylation Kit (Zymo Research). The DNA methylation status of* PRAC* was assessed by PSQ using PyroMark Q96 ID (Qiagen, Valencia, CA). The primer sequences and amplification conditions are described in Table 2. PCR reactions were conducted using 20 ng of bisulfite-converted genomic DNA. A biotin-labeled primer was used to purify the final PCR product using streptavidin-coated Sepharose beads (GE Healthcare, Buckinghamshire, UK). The PCR product was bound to Sepharose beads, purified, washed, denatured using a 0.2 mol/L NaOH solution, and washed again. Subsequently, 0.3 *μ*mol/L PSQ sequencing primer was annealed to the purified single-stranded PCR product and PSQ was performed on a PyroMark Q96 ID (Qiagen, Valencia, CA). Target CpG sites were evaluated using the instrument software (PSQ96MA 2.1, Qiagen, Valencia, CA), which converts programs to numerical values for peak heights and calculates the proportion of methylation at each base as a C/T ratio. Data analysis was performed using PyroMark Q96 ID Software v.1.0 software (Qiagen, Valencia, CA).

### 2.3. Statistical Analysis

The differences in continuous variables between two groups were assessed using a two-sample *t*-test or ANOVA trend analyses using polynomial contrasts. Median values were used as a cut-off point to divide patients into subgroups (hypomethylation or hypermethylation), and survival functions of* PRAC* genes were evaluated. The Kaplan-Meier curves were used to estimate time to recurrence or progression according to methylation status, and differences were evaluated using log-rank tests. For multivariate Cox proportional hazards regression analyses, the prognostic value of methylation status was evaluated and adjusted for well-known clinicopathological factors (sex, age, tumor size, tumor number, intravesical therapy, grade, and stage). Statistical analysis was performed using SPSS 20.0 software (IBM, Armonk, NY, USA). A *p* value <0.05 was considered statistically significant.

## 3. Results

### 3.1. Baseline Characteristics

The baseline characteristics of the NC and NMIBC patients are presented in Table 1. Mean age was 64.0 ± 13.4 years old in patients with NMIBC. Mean recurrence-free survival and progression-free survival were 47.2 ± 40.4 months (median, 35.8; range, 6.1 to 183.3) and 61.1 ± 41.7 months (median, 50.9; range, 6.6 to 183.3), respectively.

### 3.2. The Relationship between Methylation Levels and Clinicopathological Variables

As shown in Table 3, the methylation levels of* PRAC* genes were significantly higher in samples from NMIBC patients than in those from NC patients (*p* < 0.001). To evaluate the relationship between methylation patterns and clinicopathological factors, methylation levels were examined in association with well-known prognostic factors such as tumor number, size, grade, and stage. High levels of* PRAC* methylation were significantly associated with tumor grade and stage.

### 3.3. Methylation Status as a Predictor of Prognosis

**Figure 1:**
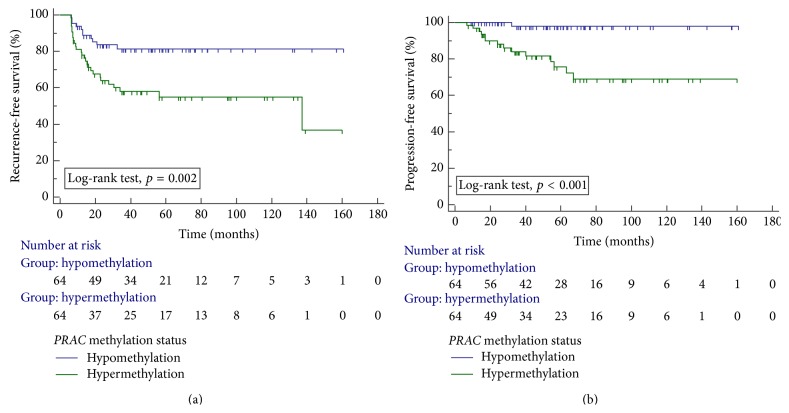
Kaplan-Meier curves of recurrence (a) and progression (b) according to methylation status of* PRAC*.

The methylation level of* PRAC* was significantly higher in the poor prognosis group (recurrence or progression) than in the favorable prognosis group (Table 3). To determine whether methylation marker was correlated with prognosis, the methylation levels of the* PRAC* gene were dichotomized (hypomethylation or hypermethylation) with the median defined as the cut-off point. Kaplan-Meier estimates revealed that the group with* PRAC* hypermethylation had significantly less time to recurrence and progression than did the* PRAC* hypomethylation group (Figure 1, log-rank test, each *p* < 0.05). Univariate and multivariate Cox regression analyses showed that* PRAC* gene methylation was an independent predictive factor of recurrence (hazard ratio [HR], 2.652; 95% confidence interval [CI], 1.241–5.667; *p* = 0.012) and progression (HR, 9.531; 95% CI, 1.172–77.497; *p* = 0.035) in patients with primary NMIBC (Table 4).

## 4. Discussion

Our results showed that methylation level of* PRAC* was significantly higher in tissues from NMIBC than in those from NC patients and that the methylation status of* PRAC* was significantly associated with higher tumor grade and advanced pathological stage. Furthermore,* PRAC* methylation status was an independent prognostic indicator of recurrence and progression in long-term follow-up of primary NMIBC patients.

Recent research investigating prognostic factors for recurrence and progression in bladder cancer has focused on epigenetic changes [3, 5, 6]. The epigenetic silencing of tumor suppressor genes may be relevant clinically because epigenetic changes may be reversible and thus restore gene function [4, 5, 7]. In transitional cell carcinoma of the bladder, several studies revealed that methylated genes such as* CDH1*,* FHIT*,* LAMC2*,* RASSF1A*,* TIMP3*,* SFRP1*,* SOX9*,* PMF1*, and* RUNX3* are associated with tumor characteristics and prognosis [3, 5, 15]. Because hypermethylation of the promoter occurs frequently in bladder cancer, detection of methylation status may be performed in exfoliated urinary cells or tumor tissues [5, 16]. Thus, markers of aberrant methylation may be a potential gateway for monitoring and determining the prognosis of bladder cancer. In the present study, the methylation of* PRAC* effectively discriminated bladder cancer from normal bladder tissues and was also associated with aggressive tumor features and poor prognosis. The results suggested that* PRAC*, a novel methylation marker identified in the present study, is specific to NMIBC and an appropriate tool to predict prognosis.

The* PRAC* gene is located on chromosome 17 at position 17q21 and is specifically expressed in prostate, rectum, and colon tissues [10]. However, little information is available about the function of the* PRAC* gene [8–12]. After the first identification of the* PRAC* gene in 2001, further research has been limited to specific organs such as prostate, rectum, and colon [11]. Recently, Arai et al. identified hypermethylation of the* PRAC* gene in RCC and showed a significant association between methylation status and prognosis [11]. These findings prompted our interest in identifying the prognostic role of* PRAC* methylation in NMIBC. To the best of our knowledge, the current study is the first to identify* PRAC* as a methylation marker related to bladder cancer.

Despite the prognostic significance of* PRAC* in NMIBC, our findings did not indicate a role in initiation or progression of bladder tumors. The lack of a clear association between these candidate markers and bladder cancer is a limitation of the present study, and this issue should be addressed in future studies. However, the objective of the present study was to identify markers related to NMIBC disease markers. Thus, we focused on the association between changes in methylation of specific markers and the associated disease phenotype rather than the effect of methylation status on gene transcription and function [4].

A key strength of our study was that we performed definitive subgroup analysis after selecting only the primary NMIBC patients. Because bladder cancer is a heterogeneous disease, and prognosis is affected by many factors, evaluating the effectiveness of a gene as a prognostic maker within a homogenous study population is important. Our findings indicate that promoter hypermethylation of* PRAC* was a reliable predictor of tumor recurrence and progression in primary NMIBC. Because methylation status was associated with an aggressive tumor phenotype, it may be used to identify patients at high risk of poor prognosis who require more aggressive treatment as well as those at low risk of recurrence and/or progression who require less intensive surveillance.

We analyzed the association between* PRAC* methylation status and prognosis in NMIBC patients. Kaplan-Meier analysis showed that NMIBC patients with hypermethylated* PRAC* had significantly decreased time to recurrence or progression. Multivariate analysis also showed that methylation of* PRAC* was an independent predictor of recurrence (HR, 2.652; *p* < 0.012) and progression (HR, 9.531; *p* < 0.035) in patients with NMIBC. Frequent recurrence is a major concern of NMIBC patients. NMIBC patients who experience progression to stage T2 during the surveillance period have a lower survival rate after cystectomy than patients who initially present with stage T2 disease [17]. Accordingly, early detection of progression to muscle invasive bladder cancer through frequent, extensive monitoring may provide a survival benefit for high-risk NMIBC patients. From a clinical point of view, epigenetic markers may be promising for early detection, prediction of treatment response, and indication of disease prognosis. The results presented herein are promising because the clinical significance was evaluated in a relatively large number of human tissue samples obtained from long-term follow-up primary NMIBC patients, and the selected methylation markers were independent predictors of disease outcome. Early cystectomy is recommended in NMIBC patients at high risk for recurrence and progression to improve survival outcomes. Therefore, our results may be useful to select the best treatment modality. However, further validation studies will be necessary to reduce false prediction rates, ensure reliable clinical relevance, and develop new therapies that target specific molecular defects, thereby reducing the morbidity associated with NMIBC.

## 5. Conclusions

Increased methylation of* PRAC* was significantly associated with a high grade and advanced stage of NMIBC. The methylation status of* PRAC* was an independent predictor of recurrence and progression in primary NMIBC. Thus, the methylation status of* PRAC* represents a useful parameter for predicting prognosis in patients with primary NMIBC.

## Figures and Tables

**Table 1:** Baseline characteristics of subjects.

Variables	NC (*n* = 8)	NMIBC (*n* = 128)
Age, yrs (mean)	59.0 ± 22.2	64 ± 13.4
Gender, number of patients (%)		
Male	6 (75.0)	107 (83.6)
Female	2 (25.0)	21 (16.4)
Number of tumors (%)		
Single	—	76 (59.4)
Multiple	—	52 (40.6)
Tumor size (%)		
<3 cm	—	76 (59.4)
≥3 cm	—	52 (40.6)
Grade, number of patients (%)		
G1	—	38 (29.7)
G2	—	72 (56.2)
G3	—	18 (14.1)
T Stage		
Ta	—	47 (36.7)
T1	—	81 (63.3)
Intravesical treatment, number of patients (%)		
No	—	58 (45.3)
Yes	—	70 (54.7)
Recurrence-free survival, months (median)		44.5 (6.2–160.7)
Recurrence, number of patients (%)		
No	—	90 (70.3)
Yes	—	38 (29.7)
Progression-free survival, months (median)		51.0 (6.6–160.7)
Progression, number of patients (%)		
No	—	113 (88.3)
Yes	—	15 (11.7)

NC: normal control; NMIBC: nonmuscle invasive bladder cancer.

**Table 2:** *PRAC* primers used for pyrosequencing analysis.

Genes	*PRAC *
Forward (5′-3′)	GGATTTTGGTTTTTATTTTTGTAGA
Reverse (5′-3′)	(biotin)-CTCACCCTTCCCTTATTTC
Sequencing primer (5′-3′)	TGTTTTTTTTTTTAATAAGGTAAAT
Amplicon location relative to TSS	−35–209
Sequence to analyze	TGATCGGTGGGCCAAGGGCGTTATCGACGGATCG
Product size	244 bp

Primer was designed using NCBI Reference Sequences build version 36.1.

The PCR reaction contained 0.01 *μ*M each of primers and Bioneer Taq (Bioneer, Daejeon, Korea), and 20 ng of bisulfite-treated DNA. The thermocycling parameters were as follows: denaturation at 94°C for 5 minutes, followed by 45 cycles at 94°C for 30 seconds, annealing at 55°C for 30 seconds, 72°C for 30 seconds, and a final extension at 72°C for 5 minutes.

bp: base pairs; *PRAC*: *prostate cancer susceptibility candidate*; TSS: transcription start site.

**Table 3:** Association between *PRAC* methylation and clinicopathological characteristics.

Variables	Methylation level (%)	*p* value
Normal versus cancer		
Normal	17.3 ± 4.0	<0.001^1^
Cancer	48.3 ± 24.3	
Number of tumors		
Single	45.6 ± 24.5	0.117^1^
Multiple	52.4 ± 23.7	
Tumor size		
<3 cm	45.4 ± 25.7	0.095^1^
≥3 cm	52.7 ± 21.7	
Grade		
G1	33.7 ± 19.4	<0.001^2^
G2	50.5 ± 23.3	
G3	70.8 ± 17.2	
T stage		
Ta	38.6 ± 24.6	<0.001^1^
T1	54.0 ± 22.4	
Recurrence		
No	43.6 ± 24.3	0.001^1^
Yes	59.6 ± 20.5	
Progression		
No	45.6 ± 23.9	<0.001^1^
Yes	69.2 ± 16.4	

^1^ *p* value calculated using Student's *t*-test.

^2^ *p* value calculated using ANOVA trend analyses test.

*PRAC*: *prostate cancer susceptibility candidate*.

**Table 4:** Multivariate Cox regression analysis of disease outcomes according to *PRAC* methylation in nonmuscle invasive bladder cancer (*n* = 128).

Variables	Recurrence			Progression		
	HR	95% CI	*p* value	HR	95% CI	*p* value
Age (<66 yrs versus ≥66 yrs)	0.711	0.364–1.390	0.319	0.884	0.310–2.519	0.817
Sex (male versus female)	1.084	0.441–2.662	0.860	1.148	0.241–5.459	0.862
Number of tumors (single versus multiple)	1.099	0.568–2.127	0.780	1.178	0.420–3.301	0.755
Tumor size (<3 cm versus ≥3 cm)	1.270	0.645–2.499	0.490	4.149	1.184–14.540	0.026
T Stage (Ta versus T1)	0.963	0.437–2.122	0.925	2.028	0.233–17.623	0.521
Grade (G1-2 versus G3)			0.493			0.021
G1	1	—	—	1	—	—
G2	1.124	0.450–2.811	0.803	1.235	0.074–20.544	0.883
G3	1.825	0.567–5.879	0.313	7.280	0.397–133.445	0.181
Intravesical therapy (no versus yes)	1.248	0.610–2.554	0.545	2.061	0.558–7.612	0.558
*PRAC* (hypomethylation versus hypermethylation)	2.652	1.241–5.667	0.012	9.531	1.172–77.497	0.035

HR: hazard ratio; CI: confidence interval; *PRAC*: *prostate cancer susceptibility candidate*.
